# Hydraulic fracturing: New uncertainty based modeling approach for process design using Monte Carlo simulation technique

**DOI:** 10.1371/journal.pone.0236726

**Published:** 2020-07-29

**Authors:** Awad Ahmed Quosay, Dariusz Knez, Jan Ziaja

**Affiliations:** 1 Department of Petroleum Engineering, University of Khartoum, Khartoum, Sudan; 2 Faculty of Drilling, Oil and Gas, AGH University of Science and Technology, Krakow, Poland; China University of Mining and Technology, CHINA

## Abstract

Hydraulic fracturing is a key method used in completion of shale gas wells as well as in well stimulation. There are a lot of factors affecting the hydraulic fracturing treatment; i.e. formation in-situ stresses, fracturing fluid properties, proppant, pumping rate, reservoir fluid and rock properties…etc. For predictive modeling, these factors are associated with a lot of uncertainties, since most of them are laboratory measured, calculated or subjectively estimated. Moreover, the precise contribution of each factor on the final fracturing result is unknown for each individual case. Therefore, for better treatment performance and in order to find the best range of designing parameters, a hydraulic fracturing predictive model that involves these uncertainties is required specially for newly exploited shale gas reservoir. In this paper a new uncertainty-based approach is described for hydraulic fracturing processes. It is based on assigning probability distribution for some variables and parameters used in the designing process. These probability distributions are used as input data for analytical equations that describe the fracturing processes. Monte Carlo Simulation technique is used to apply uncertainty-based values on the designing analytical formulas. A hypothetical hydraulic fracturing example is used to simulate the effect of different variables and designing parameters on the entire fracturing process. The simulation results are illustrated into probability distribution curves and variance-based sensitivity analysis is performed to assess the contribution and the correlation between different variables and outcomes. Fracture geometry is almost controlled by the injection fluid’s viscosity, in case of constant injection rate; while rock properties have insignificant effect on the fracture width compares to fracturing fluid’s effect. Therefore more emphases shall be directed to rheological modeling of the fracturing fluid. It is found also that fracture height, which is difficult to be estimated, is the most crucial parameter in the calculation of treatment size or the injected fluid’s volume. Proppant porosity, injected fluid viscosity and formation strength are slightly affecting propped fracture width, while proppant final concentration plays the main role of determining the calculated propped fracture width. It is observed from the simulation results that the initial formation permeability will extremely affect the post fracturing skin factor while other formation rock properties have almost no effect on the skin factor. Throughout the implementation of the uncertainty-based modeling approach for hydraulic fracturing process design, it is found that uncertainties in the value of many variables and parameters are slightly affecting the process outcomes. However, injected fluid viscosity, shale formation permeability and proppant final concentration are found to be the most influencing factors in the entire process. Therefore, it is highly recommended to perform in-depth study for these factors prior conducting any designing process of hydraulic fracturing.

## 1. Introduction

Hydraulic fracturing (HF) techniques are particularly effective in stimulating hydrocarbon production from shale gas or oil formations. Since ultra-low permeability coefficients of shale formation make it hard for hydrocarbons to transport towards wellbore, therefore artificial fractures are induced to the formation in order to achieve commercial gas production rate. Directional and horizontal drilling combined with hydraulic fracturing have made the production of natural gas from different shale formations achievable in some parts of the world.

Hydraulic fractures are created by the injected fluid under high pressure, such as slick water. The fluid that is used in HF is mixed with proppants, such as particles of sand. The proppant is pumped into the fractured rock in order to help keeping the fractures relatively permeable to formation fluids once the fracturing pressure from the injected fluid is released. In addition to the fracturing fluid and the proppant, some other chemicals are added to the fluid mixture. These chemicals can serve many functions in HF, specially controlling the injected fluid’s viscosity.

Computational models for HF treatment are the base of the design process. Quantification of risks and opportunities associated with the fracturing job will lead to making the right decision [1].

For instance, in tight gas sand wells, the following designing and operational factors are highly affecting the overall performance of the HF treatment outputs:

- gas reservoir characteristics, i.e. gas saturation, net pay zone thickness.etc.
- injected water quality: bicarbonate content and the peak viscosity,
- amount of proppant injected into the fracture.

It was observed from analyzing a set of hydraulic fractured well’s data that placing more into the fracture will not bring significant increase in post treatment production rate. It was found in the literature that higher post treatment production rate can be achieved for low viscosity fracturing fluids. Therefore, there is no need for high viscosity fluids [2]. Therefore, although a lot of sophisticated software and programs were developed to design most of the HF treatments carried on oil and gas wells but still contradicting results and sometimes unfavorable outcomes are occurring. This is mainly due to the following reasons:

1. Uncertainties associated with the input data into Software/programs used for treatment design
2. Cost of acquiring logs and other technical data is not proportional with the technical value of information/data. Therefore the value of information must be quantified and properly ranked.
3. In many cases decision process is not based on probability assessment of each and every factor affecting the treatment design.

There are numerus papers presenting influence of chosen parameters on fracture propagation simulation results [3–5] but so far there is no complex uncertainty based analysis. Analytical models describe influence of many parameters on the initiation pressure in perforations [6] as well as on fracturing pressure [7]. Comparison of computer simulation results and data from fracture monitoring shows high complexity of investigated problem and uncertainty in development solutions [8]. Some applications of reservoir and economic uncertainty shows strong influence on the fracture design [9]. The main objective of this paper is to identify and to quantify the effect of uncertainties of different variables and parameters in the design outcomes of HF treatment. Classical models are still base for more sophisticated simulators. Authors decided to use classical approach to avoid influence of complexity factor on results. If the impact of model’s input data quality is quantified then this will save a lot of resources, efforts and time for future designs of HF treatments for both operating and service companies.

## 2. Methods and tools

Successful HF job requires accurate treatment design, while the design must be carried on using data set (valuable information) with high level of accuracy (at least for the key variables). Therefore, these key variables must be identified and ranked according to their contribution to the final treatment outcomes in order to reduce the costs of obtaining this valuable information. Our main objectives will be achieved through:

1. Process description of the HF treatment and how the probability based model of the treatment can assist decision makers.
2. Development of a simple computational model to simulate HF treatment in a vertical well
3. Application of variance-based sensitivity analysis (VBSA) by utilizing Monte Carlo Simulation that is ran on the developed probability based model.
4. Ranking of the model’s input variables in term of their impacts on the HF design outputs

In order to help analyzing HF treatment decision, results of sensitivity analysis should be quantitative rather than qualitative to enable meaningful comparisons between the uncertainties of the inputs and its impact on the same output. Therefore the desired sensitivity analysis method should be independent of model assumptions [10]. Variance-based sensitivity analysis (VBSA) is a form of global sensitivity analysis, in which the “variance” measures how far a set of numbers are spread out around the average value of a variable [11]. When a certain problem is modeled in a probabilistic framework; i.e. inputs are given in terms of ranges or probability distribution curves, VBSA decomposes the variance of the output of the model into fractions or percentages which can be attributed to inputs [12]. The VBSA can be applied through the following steps:

- building a computational model and defining its input and output variable(s),
- defining uncertainty ranges and assigning probability density functions to each input variable,
- using random sampling method (as for Monte Carlo Simulation) and generating an input matrix and, evaluating the output, and
- assessing the influences of each input variable on the model output

Once a certain formation or well is exposed to HF operation the next step will be gathering data. Usually the collected formation data is always associated with lots of uncertainties. Hence, acquiring more information will cost time, effort and resources [13]. The uncertainties in reservoir formation data usually occurs due to the following reasons:

- Having a single point value for the number of reservoir parameters and factors
- Subjective estimation for missing information or data gaps
- Errors and variances in measurements and readings in previous data sets
- Miscalculating based background assumptions especially when using commercial software.
- Time factor: values a lot of reservoir and well-flowing parameters are time-function
- Others, such as human and software errors, …, etc.

Thus, it is important to evaluate the risk of using uncertain values for some of the variables and parameters used during the screening or designing of a HF process and to answer the question of how to accommodate uncertainties in the required data for HF design and operation.

### 2.1 Process description

**Fig 1:**
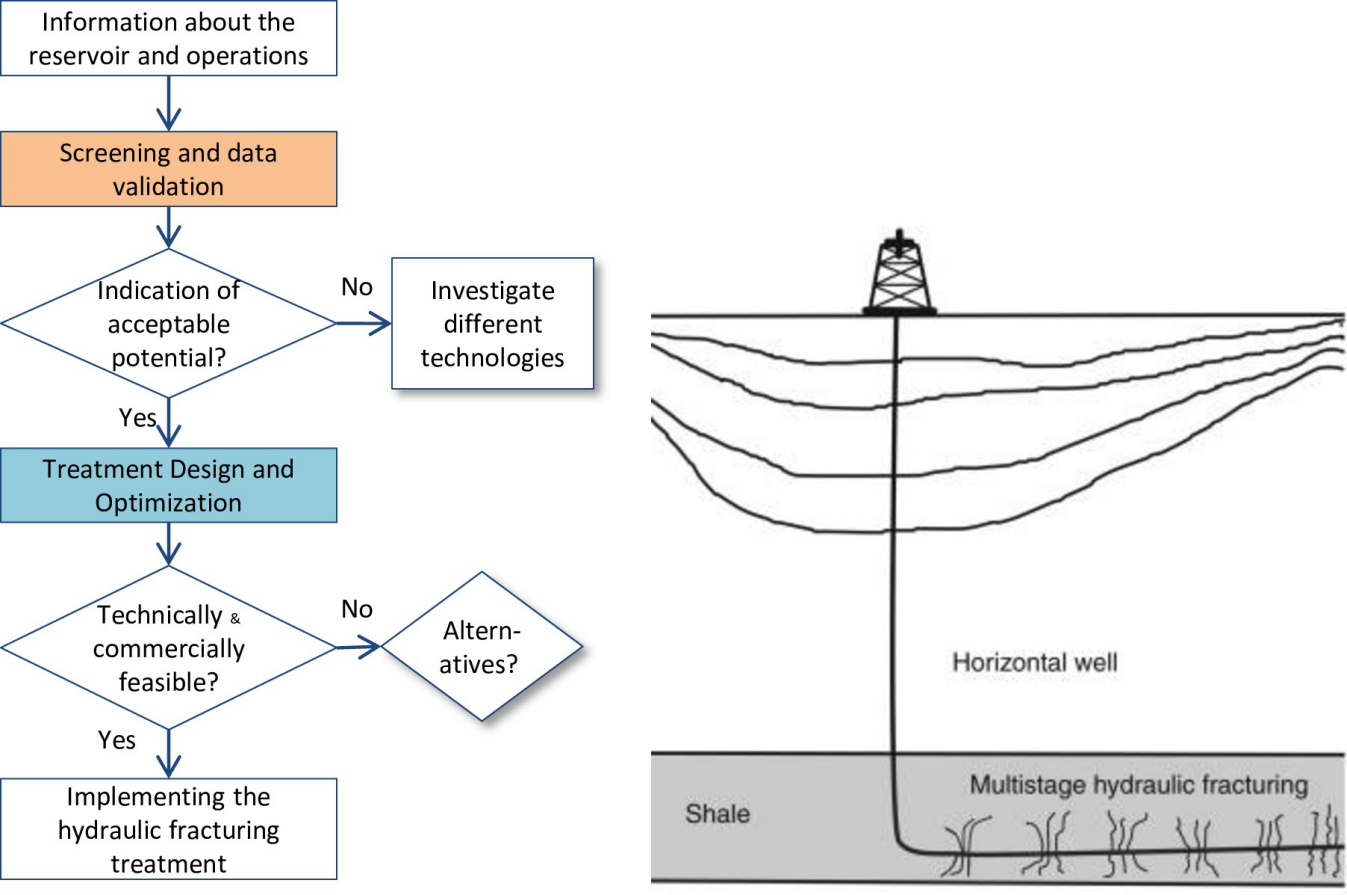
Process flow for a hydraulic fracturing treatment.

The decision of undertaking any well stimulation treatment such as, hydraulic fracturing treatment is usually goes through several steps; starts by performing general screening process and end up with implementing the treatment’s operation at well site. Fig 1 illustrates the process flow for a hydraulic fracturing treatment with explanatory figure of the gas production in shale reservoirs with multi-staged hydraulic fracturing technology [14]. The crucial technical decisions in this process are made after the application of two steps: screening and process design and optimization.

The screening step includes data collection, validation and application on the principle equations for hydraulic fracturing treatment design. While the treatment design step involves tuning of design parameters to achieve optimum forecasted outcomes of the proposed treatment. Both steps, i.e. screening and treatment design are conducted through the application of governing equations, i.e. analytical, imperial or numerical formulas which calculate and predict:

1. Breakdown pressure and fracture’s geometry,
2. injected fluid volume, leak-off volume and fracture volume,
3. proppant concentration and mixing schedule, and
4. post-fracturing skin factor.

Since hydraulic fracturing process design is a combination of lots of data, technical constrains and series of equations; and one can say that the success or failure of a fracturing job can be traced to both the availability and the accuracy of data/information necessary for optimizing the process design [15]. And since, for new fields, information about the reservoir properties are usually limited, as well as the operational parameters of fracturing process are not precisely defined; therefore, the technical variables and calculated parameters that are used in the fracturing process design, such as fractures shape and geometry, injected fluid volume, proppant concentration and pumping schedule are having wide range of uncertainties. That is because, appropriate operational process design requires the knowledge of various reservoir rock and fluid properties as well as deep understanding of equations governing the injection and fracturing processes.

Commercial hydraulic fracturing simulators are often used to design the fracturing process [16]. In order to use these simulators in efficient way, considerable amount of datasets as well as laboratory tests are required. Reservoir data necessary for the simulation include reservoir rock and fluid properties; such as pore pressure, formation tectonic stresses, Young’s modulus, Biot’s poroelastic constant, average density of overburden rocks, Poison’s ratio, porosity, formation permeability, layer thickness, …etc. Data set of injected fracturing fluid’s properties and well construction are also required for the simulation inputs. Therefore in order to obtain good simulation outcomes with reasonable accuracy, the abovementioned data together with the reservoir geological model shall be available and validated [17]. However, high uncertainties in the simulation input data will result in wrong predictions and therefore undesirable outcomes can occur. This work investigates the influence of input parameters uncertainties on process design using Monte Carlo simulation technique. Research shows that data validation is very important and gives direct benefits for petroleum industry.

## 3. Uncertainty based screening and designing method for hydraulic fracturing treatment

In this paper, description of a new designing approach for hydraulic fracturing is introduced. This new approach is based on assigning different probability distributions for some variables and parameters used in the screening and designing processes of hydraulic fracturing. These uncertainty-based values are used as input data for analytical equations that describe the fracturing processes. Monte Carlo Simulation technique is used to apply the designing analytical formulas on the uncertainty-based values. Monte Carlo simulation is a numerical modeling technique, named after the city of Monte Carlo in Monaco. It is a technique that uses a random number generator to produce and extract an uncertain variable within a distribution model for calculation in a given formula or correlation. The main target of this approach is to identify the most important factors that could highly affect the hydraulic fracturing job and then consequently the risk of applying uncertainties data will be mitigated. The details for the attentive steps in Fig 1 are as follows.

- Screening and data validation step:
  1. determine the common formulas and equations to be used in the design processes,
  2. define possible ranges for design parameters and variables with probability distribution curves,
  3. apply Monte Carlo Simulation on the process design calculations,
  4. display the calculated results for each stage in probability distribution curve with acceptable confidence intervals,
  5. perform sensitivity analysis on the calculation’s outcomes,
  6. screen out the most influencing parameters and variables on the calculated outcomes,
  7. apply more investigations on the defined possible ranges and probability distributions curves of the screened parameters,
  8. re-do the treatment design processes with narrower ranges and more accurate values of the parameters and present the outcomes in uncertainty based format,
  9. evaluate the possible outcomes of the treatment (technical and commercial).
- Decision Making Process. To apply decision analysis process on the following alternatives:
  1. to perform the treatment design,
  2. to wait,
  3. not to apply the treatment.
- Treatment Design and Optimization
  1. define the constrains and boundary conditions/ limits,
  2. define decision parameters and their possible ranges,
  3. apply Monte Carlo simulation on design process with the defined decision parameters to calculate their optimum values,
  4. cross check the calculation outcomes with the outcomes of commercial software(s) simulation.

In this paper the items from 1 to 6 in the screening step will be demonstrated for hydraulic fracturing treatment. Therefore, a hypothetical hydraulic fracturing example was generated to illustrate the effect of different designing variables on each sub-division of the fracturing process. Monte Carlo simulation technique is used to assign probability distribution for the algorithm build by authors. The simulation results were illustrated and sensitivity analysis is performed.

## 4. Modeling fracture geometry

In our case it is important to reflect the effect of different parameters statistically on the fracture average width.

The following three models assume that the fracture is planar, that is, fracture propagates in a particular direction [15].

- **Radial Fracture Model**
  A simple radial (penny-shaped) crack/fracture model was first presented by Sneddon and Elliot [18].
  Geertsma and de Klerk [19] applied modification on the model where the average fracture width is given by:
  $$w_{\mathrm{avg}}=0.279{\left[\frac{\mu \cdot q_{i}\cdot (1-\nu )\cdot R}{E}\right]}^{\frac{1}{4}}$$ (1)
  where:
  w_avg_ = average fracture width, m
  μ = fluid viscosity, Pa.s
  q_i_ = pumping rate, L/s
  ν = Poisson’s ratio, -
  R = the radius of the fracture, m
  E = Young’s modulus, MPa
- **The KGD Model (**Khristianovich-Geertsma-DeKlerk)
  The model is based on the following assumptions:
  - fracture width is proportional to fracture length,
  - the pressure within the fracture will decline as fracture propagates.
    $$w_{\mathrm{avg}}=0.075{\left[\frac{\mu \cdot q_{i}\cdot (1-\nu )\cdot x_{f}^{2}}{{\mathrm{Gh}}_{f}}\right]}^{\frac{1}{4}}\cdot \left(\frac{\pi }{4}\right)$$ (2)
    where:
    G = E / 2(1 + υ), shear modulus, MPa
    h_f_ = fracture height, m
    x_f_ = fracture half length, m
- **The PKN model (**Perkins-Kern Nordgren)
  The model is based on the following assumptions:
  - fracture width is proportional to fracture height,
  - with the fracture propagation the pressure within the fracture will decrease.
    $$w_{\mathrm{avg}}=0.099{\left[\frac{\mu \cdot q_{i}\cdot (1-\nu )\cdot x_{f}}{G}\right]}^{\frac{1}{4}}\cdot \left(\frac{\pi }{4}\cdot \gamma \right)$$ (3)
    Where γ ≈ 0.75 (a constant describes the pressure gradient within the fracture based on fluid rheology)
    Note: the PKN solution is only valid when the fracture length is at least three times the height.

Since fracture width is a key designing parameter for hydraulic fracturing design, a hypothetical example is modeled in the same way it was done for breakdown pressure and surface injection pressure. The model is based on the following assumptions:

1. injected fluid viscosity has got high possibility to change during the process,
2. fracture width calculation is based on fracture half length of 137m as the design objective,
3. fracturing fluid injection rate is assumed to be a designing parameter, i.e. a variable with uniform probability distribution, which can be optimized in the designing and optimization step.

The three models were simulated according to Eqs (1), (2) and (3) using Monte Carlo simulation technique.

While all variables are given the same degree of uncertainty, e.g. ±20%, which is not realistic since the uncertainty degree in fluid properties cannot be similar to as it is in rock properties; it was observed from the simulation results that the calculated fracture width for the three said models is very much depending on the injection flow rate.

However in a second trial, a constant injection rate of 106 l/s is assumed and a sound realistic degree of uncertainty is given for each variable as shown in Table 1.

**Table 1:** Assumptions for calculating fracture width according to the common analytical models.

#	Variable	Min.	Mean	Max.	Probability Distribution type	Main source of uncertainties	Degree of uncertainty	
1	Viscosity, Pa.s*	0.001	0.005	0.0095	Triangular	Effect of formation temperature and degradation	High	~±80%
3	Young's Modulus, MPa	49,680	54,855	60,030	Triangular	Formation complexity	Low	~±10%
4	Poison’s Ratio	0.28	0.355	0.43	Triangular	Formation complexity	Medium	~±20%
5	Fracture Height, m	24	34	44	Triangular	Rock heterogeneity	Medium	±30%

* The range of injected fluid viscosity is assumed according to a new trend of low viscosity hydraulic fracturing fluid, while the viscosity on the surface (before injection) is just below 10 cp.

A quick summary of the simulation result is shown in the Table 2. Values in the Table 2 were obtained applying data of Table 1 in Eqs (1), (2) and (3) with the aid of Monte Carlo simulation, that is giving results after 30,000s of trials using all probability density functions given for each parameter in the equations.

**Table 2:** Comparison between the calculated fracture’s average width based on the three analytical models.

Statistics	Fracture average width, [mm]		
	Radial Model	KGD Model	PKN Model
Mean	27.24	10.55	7.29
Standard Deviation	2.34	0.90	0.62
Minimum	18.57	7.23	4.99
Maximum	32.45	12.49	8.63

**Fig 2:**
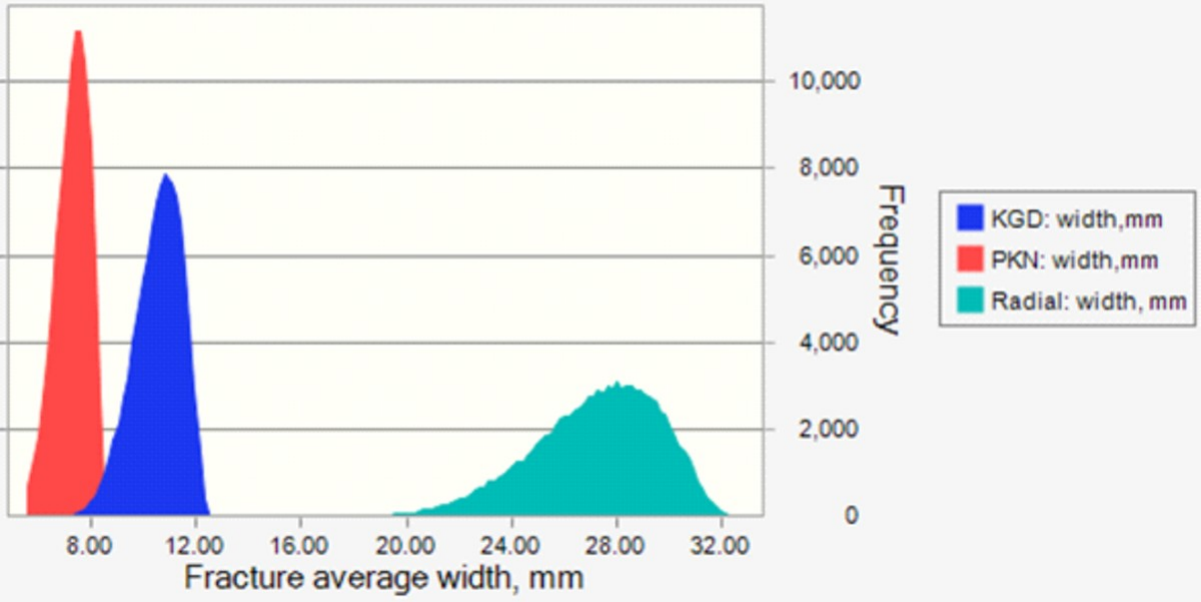
Fracture average width calculated for Radial, KGD and PKN models.

It is obvious that the three models give very different values for the fracture average width that is because of each model is based on different assumptions. Fig 2 shows the resulted probability distribution for each model in term of frequency of (probability density function) versus the fracture average width, i.e. it basically gives a description of how the entire population from which the calculated data is drawn is spread out over different values of fracture’s average width. The Fig 2 illustrates that there is minimal intersection between KGD and PKN models though the values calculated based on radial model are far bigger than KGD’s and PKN’s. From the sensitivity analysis, it is found that the fracturing fluid’s viscosity and injection rate have the highest contribution on the calculated fracture average width.

The simulation results show that the three models are giving very different ranges of fracture’s average width. According to this example, radial model gives almost four times larger fracture width than it is calculated by PKN model. Although KGD model is giving moderate values for the fracture width but it depends on additional assumption which is fracture height. According to the sensitivity analysis that is performed on the three models, the calculated fracture width is very much depending on the injected fluid viscosity. Higher injection rate will also affect the fracture width positively.

As a conclusion, fracture geometry is almost controlled by the injection’s fluid viscosity in case of constant injection rate during the process. Therefore predicted fracture width magnitude is considered to be a strong function of fluid properties with slight dependency on rock properties, i.e. Young’s Modulus and Poisson’s ratio. Accordingly, it is recommended to put more emphases on rheological modeling of fracturing fluid. It can be concluded that injected fluid viscosity is the main designing factor that determine fracture geometry.

## 5. Injection, leak-off and fracture volumes

Basically fracture geometry determines treatment size, i.e. injected fluid volume and proppant volume. However since part of the fracturing fluid is going to leak off into the formation, therefore the total injected fluid has to be more than the fracture’s volume. In this section, treatment size and injection time are modeled using KGD fracture model for simplicity, since the main target is to define the contribution of each variable on the final outcomes, i.e. injection fluid volume *V*_*inj*_, leak-off volume *V*_*leak*_, fracture volume *V*_*f*_ and injection time *t*_*i*._

Based on material balance, volumes are related as [16]:

$$V_{\mathrm{inj}}=V_{f}+V_{\mathrm{leak}}$$ (4)

Where:

$$V_{inj}=q_{i}\cdot t_{i}$$ (5)

$$V_{f}=2\cdot x_{f}\cdot h_{f}\cdot w_{avg}$$ (6)

$$V_{\mathrm{leak}}=2\pi \cdot C_{L}\cdot \left(x_{f}\cdot h_{f}\right)\cdot r_{p}\cdot \sqrt{t_{i}}$$ (7)

In where:

C_L_= leak-off coefficient [m/sec^0.5^]

r_p_ = the ratio between fracture area and leak-off area

Data from the previous examples are taken together with the variables that were described in the above equations {from Eqs (4) to (7)}, where:

- C_L_ is given a range from: 0.39×10^−4^ to 0.118× 10^−3^ m/s^0.5^, and
- r_p_ is given a wide range from 0.42 up to 0.98

Since both V_inj_ and V_leak_, are depending on injection time, which is not known in the beginning, a numerical iteration procedure was performed, where initial value of injection time is assumed to carry on the calculation and then it is corrected during the iterations [15].

**Fig 3:**
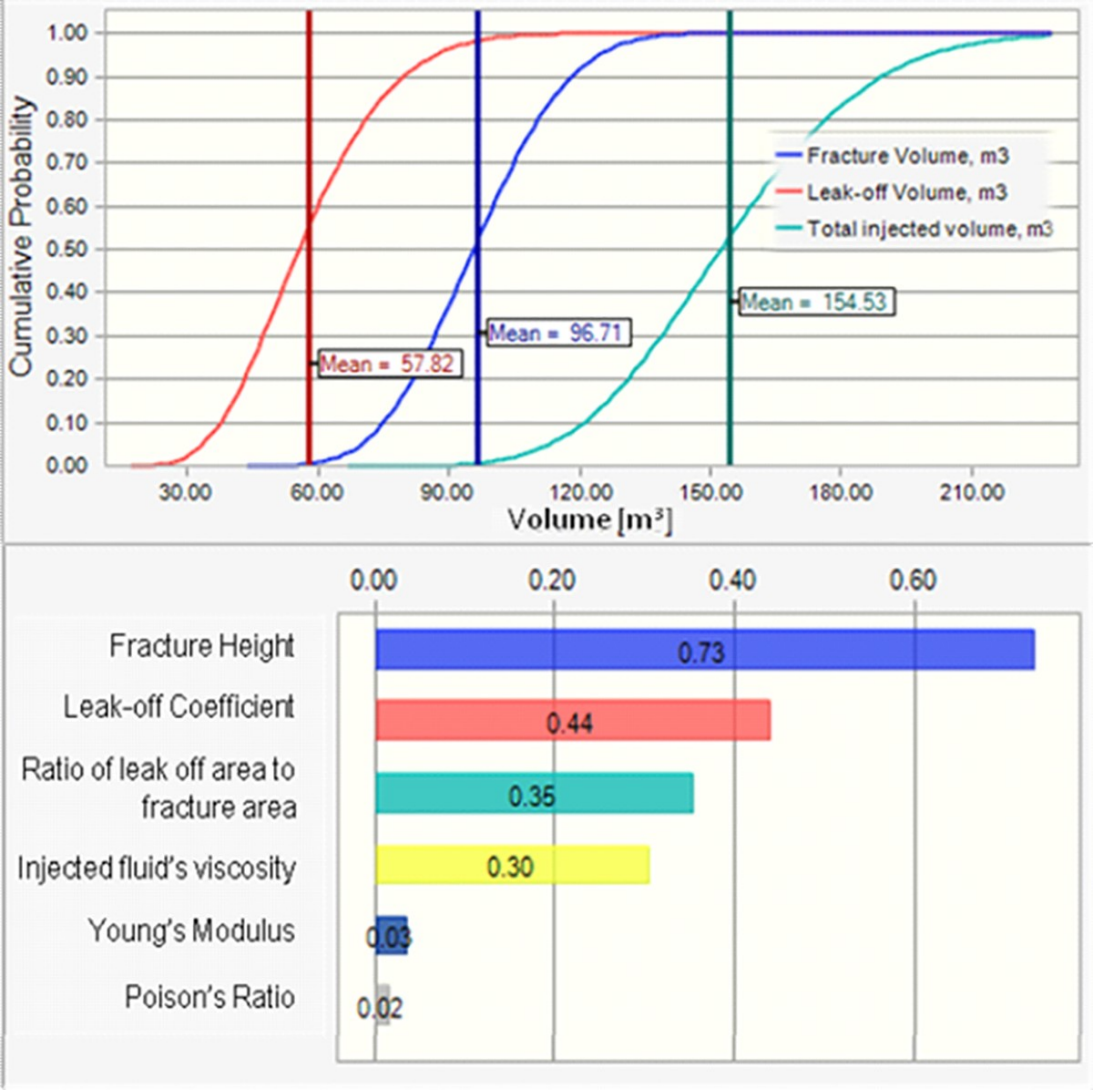
Fracture, leak-off and total injection fluid estimated volumes and the relationship correlations between different variables and the total injected fluid’s volume*. * The correlation coefficient is estimated by ordering values of each variable from the lowest to the highest (regardless its probability distribution functions) and correlate these values with the outcome values (in this case is the Total Injected Fluid Volume); then the correlation coefficient is calculated in which:- A correlation of 1 indicates a perfect positive correlation, minus 1 indicates a perfect negative correlation, and 0 indicates there is no correlation between the variable and the outcome.

The simulation results (as cumulative probability chart for fracture volume, leak-off volume and total injected fluid’s volume) and sensitivities are shown in the Fig 3, where the summation of mean values of fracture’s volume and leak-off volume is nearly equal to total injected volume.

It is found that fracture height is the most crucial parameter in the calculation of treatment size, in where it is proportional to fracture size/volume and inverse proportional to leak-off volume. Injected fluid viscosity has a considerable effect (positively and negatively) on fracture volume and leak-off volume, respectively. Total injected volume is found to be very sensitive to variations in fracture’s height and less sensitive to variations in leak-off coefficient and injected fluid’s viscosity. On the other hand, there is strong proportional relationship between the injected fluid’s volume with fracture’s height with correlation coefficient ~73% as it is shown in Fig 3.

## 6. Calculation of proppant’s concentration

Generally proppants that are placed into fractures should be permeable to formation fluids under high pressures; the gap between particles should be adequately large. The proppant must have the mechanical strength to withstand closure stresses to hold fractures open after the fracturing pressure is released [20]. In general, larger diameter proppant yields better permeability, but proppant size must be checked against proppant admittance criteria through the perforations and inside the fracture.

Pad volume and proppant concentration determination is an important step during hydraulic fracturing design process. Insufficient pad volume results in premature screen-out [16].

The pad volume, V_pad_ is calculated as:

$$V_{pad}=V_{inj}\cdot \varepsilon$$ (8)

Where

$$\varepsilon =\frac{1-\psi }{1+\psi }$$ (9)

$$\psi =\frac{V_{f}}{V_{\mathrm{inj}}}$$ (10)

In which, Ɛ is a dimensionless number represents proppant volume fraction, while Ψ is the ratio between fracture volume and total injected volume or in other word the “injected fluid efficiency”.

On the other hand, the proppant concentration/mixing schedule, c_p_(t), is found by:

$$c_{p}(t)=c_{f}{\left(\frac{t-t_{pad}}{t_{i}-t_{pad}}\right)}^{\varepsilon }$$ (11)

where *c*_*f*_ is the final proppant concentration in kilogram per liter (kg/L) and *t*_*i*_ is the injection time. The time required to pump the pad volume at a known injection rate, *t*_*pad*_, is found by:

$$t_{\mathrm{pad}}=\frac{V_{\mathrm{pad}}}{q_{i}}$$ (12)

Finally, predicting propped fracture width (w_p_) can be obtained through the following equation:

$$w_{p}=\frac{C_{p}}{\left(1-\phi_{p}\right)\cdot \rho_{p}}$$ (13)

where

ϕ_p_ = proppant porosity,-,

ρ_p_ = proppant density [kg/m^3^],

$$C_{p}=\frac{M_{p}}{2\cdot x_{f}\cdot h_{f}}$$ (14)

and the proppant weight M_p_ is found by:

$$M_{p}=\frac{c_{f}}{1+\varepsilon }\cdot \left(V_{inj}-V_{pad}\right)$$ (15)

In order to perform uncertainty-based calculation for proppant concentration schedule and the final propped width, the following assumptions are considered:

- proppant density as a single value variable = 2643kg/m^3^,
- proppant porosity is ranged between 0.25 to 0.45 with uniform probability distribution,
- proppant final concentration is ranged between 0.24 to 0.48kg/L with also a uniform probability distribution. This factor is a designing parameter that requires optimization.

**Fig 4:**
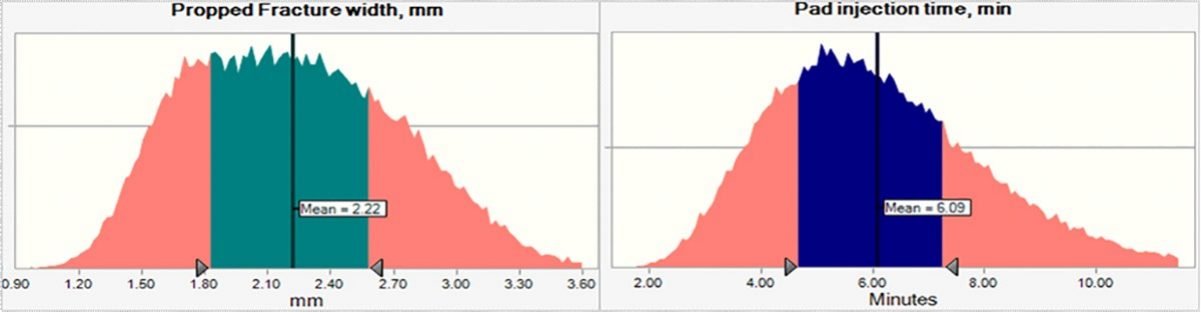
Calculated propped fracture width and pad injection time.

Monte Carlo simulation is performed on Eqs (8) to (15) based on the same data set used earlier. The calculations outcomes for both pad volume and propped width are demonstrated in Fig 4.

The propped fracture width is found to be much less in magnitude than the fracture width calculated based on KGD model. The mean value of the KGD fracture width is equals ~ 5 times the propped width in this example. On the other hand, the propped fracture width is found to be a strong function of proppant final concentration. While injected fluid viscosity and proppant porosity are also affecting the calculated propped fracture width in much lower scale (Table 3).

**Table 3:** Contribution and correlation of different variables to the propped fracture width.

Assumptions	Contribution	Correlation
Proppant final concentration, [kg/L]	78%	0.87
Injected fluid viscosity, [Pa·s]	12%	0.35
Proppant Porosity	7%	0.27
Fracture Height, [m]	2%	-0.14
Other variables	1%	

**Fig 5:**
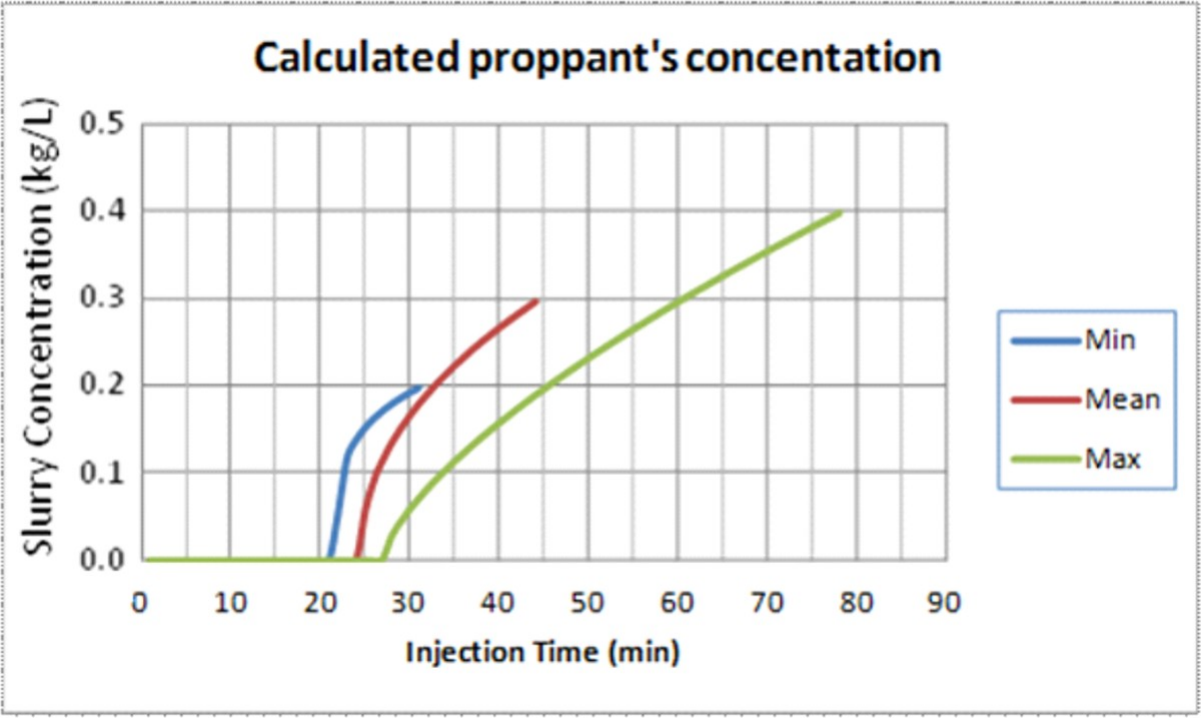
Proppant mixing schedule calculated for different ranges of pad injection time and proppant final concentrations.

The proppant mixing schedule is calculated for the minimum, mean and maximum values of the assumed variables. Fig 4 shows the slurry concentration in (kg/L) during the injection time. The minimum, mean and maximum values of the pad injection time, as shown in Fig 5, are considered the initial points to calculate the proppant concentration schedule. It is observed that the final proppant concentration is playing a main role in the slurry injection schedule.

## 7. Post-fracturing skin factor

Productivity of fractured well depends on fracture dimension (length and height) as well as fracture permeability, which is the main factor controlling transportation of reservoir’s fluid to the wellbore. Argawal et al. [21] and Cinco-Ley and Samaniego [22], defined the fracture conductivity as:

$$F_{\mathrm{CD}}=\frac{k_{f}\cdot w}{k\cdot x_{f}}$$ (16)

where

*F*_*CD*_ = fracture conductivity, dimensionless

*k*_*f*_ and *k* are fracture permeability and formation permeability, respectively; measured in [mD]

*w* and *x*_*f*_ are fracture width and half-length in meter.

Usually proppant selection will be based on its compressive strength that can overcome the formation closure stress during the production stage. Closure stress (σ_c_) is the pressure at which the fracture closes after the fracturing pressure is relaxed. It is usually between 80% and 90% of breakdown pressure [23]. During back flow of reservoir fluid into wellbore, higher pressure drawdown means less bottom hole flowing pressure. Consequently pore pressure around the wellbore will decrease and then the effective horizontal stress will increase. The following equation indicates that less pore pressure will lead to more effective horizontal stress [15], σ^`^_h_:

$$\sigma_{c}={\sigma '}_{h}=\frac{\nu }{1-\nu }\cdot \left(\rho \cdot g\cdot H-\alpha \cdot P_{p}\right)$$ (17)

For approximation, the permeability of various types of proppants under different fracture closure stress can be formulated as:

$$log\;k_{f}\approx a-b\;\sigma_{c}$$ (18)

Where (a) ranges from 300 to 550 Darcy, and (b) ranges from 0.018 to 0.024 Darcy/MPa. (a) and (b) are described hereinafter as proppant’s type factors. This was modeled based on data published by Economides and Nolte [16].

Once fracture permeability is estimated then post-treatment skin factor (s_f_) can be calculated throughout the relationship between fracture conductivity F_CD_ and the term s_f_ +ln(x_f_/r_w_) [23]:

$$s_{f}+\ln\left(\frac{x_{f}}{r_{W}}\right)=\frac{1.65-0.328\ln\left(F_{CD}\right)+0.116\cdot {\left(\ln\left(F_{CD}\right)\right)}^{2}}{1+0.18\cdot \ln\left(F_{CD}\right)+0.064\cdot {\left(\ln\left(F_{CD}\right)\right)}^{2}+0.05\cdot {\left(\ln\left(F_{CD}\right)\right)}^{3}}$$ (19)

Monte Carlo simulation is applied on the Eqs set: (16) to (19), in where the following assumptions are considered:

- shale gas formation permeability is assumed between 0.0001 to 0.05 mD, with uniform probability distribution.
- wellbore radius is assumed ~0.143 m as a single data value,
- bottom hole flowing pressure is given a range between 5.4 to 13.6MPa, and
- fracture half length is given a range between 100m to 400m with uniform distribution

**Fig 6:**
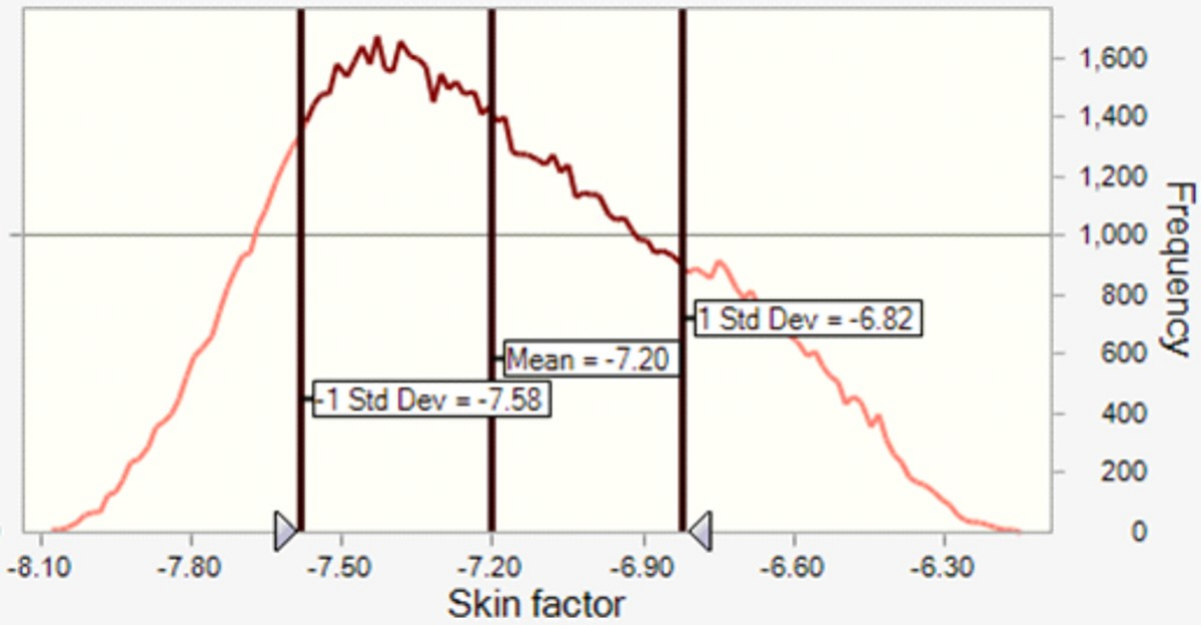
Calculated post-fracturing skin factor.

The calculation result for the post fracturing skin factor is shown in Fig 6.

The calculated skin factor has negative values (less than -7) which indicates that fracturing in shale formation that have uncertain rock properties, stress profiles, fracturing geometry and injected fluid properties can still create negative skin factor.

Sensitivity analysis is applied for the post-treatment skin factor and it is observed that the uncertainty in initial formation permeability will extremely affect the calculated skin factor case the designed fracture half length can be achieved with minimal uncertainty, otherwise for uncertain range of possible fracture length, then post treatment skin factor will be mostly vulnerable by the magnitude of fracture’s half length. On the other hand, proppant type, injected fluid viscosity and formation rock properties have minor effect on the calculated skin factor (Table 4).

**Table 4:** Sensitivity analysis on the post-fracturing skin factor.

Assumptions	Contribution	Correlation
Fracture Half length, [m]	83.87%	-0.9
Formation permeability, [D]	13.07%	0.36
Proppant’s type factor	1.41%	-0.12
Proppant final concentration, [kg/L]	1.18%	-0.11
Injected fluid viscosity, [Pa·s]	0.22%	-0.05
Proppant Porosity	0.09%	-0.03
Poison's Ratio, υ	0.09%	0.03

## 8. Uncertainty-based model for hydraulic fracturing designing process

Usually analytical model has less input data than the 2D or 3D commercial fracturing simulators. Although this can be considered as an advantage for the analytical model but on the other hand the sophisticated commercial numerical models are supposed to be more accurate since they consider more variables, which again lead to the dilemma of data uncertainties. However, it is found throughout this study that the uncertainty of many variables and parameters are not really affecting the process design outcomes and vice versa. Therefore it is very important to validate and to assure the design data quality, specially for the factors that highly influencing the process outcomes.

Hence, collective statistical model (or uncertainty based model) is required to enhance the quality of the analytical model.

From the previous analysis using Monte Carlo simulation techniques on the basic analytical equations that are used to design hydraulic fracturing process, it is found that some of the variables used in some particular equations can affect the outcome of other equations however there are no direct relationship with these variables. For example injected fluid viscosity and Young’s modulus collectively have a significant effect on the value of fracture leak off volume although according to Eq (7), there is no direct relationship between them. On the other hand, statistically, through variance based sensitivity analysis technique, it is found that some variables have almost minor effect on the outcomes nevertheless a mathematical formula is valid. Throughout the implementation of the uncertainty-based modeling approach for hydraulic fracturing process design, it is found that some of the model’s input variables as shown in the Table 5, are the most influencing parameters in the entire hydraulic fracturing process. This was obtained when +/- 10% error is given to all the variables in the model as upper and lower limits in triangular probability distribution.

**Table 5:** Summary of the most influencing variables for some of the hydraulic fracturing model’s outcomes.

Model’s outputs Input variables*	Injection pressure	Injected fluid’s volume	Proppant placement	Post fracturing skin factor
Tubing length				
Tubing inner diameter				
Specific gravity of injected fluid				
Wellbore radius				
Formation depth				
Pore pressure gradient				
Poison's ratio	High			
Average density of overburden	High	High		
Biot's poroelastic constant				
Tectonic stress				
Tensile strength of the rock				
Injected fluid viscosity				
Pay zone thickness		High		
Young's modulus				
Fracture half length				
Fracture height				
Ratio of leak off area to fracture area			High	
Leak off coefficient			High	
Proppant porosity				
Proppant final concentration				
Proppant type—(a) factor, k vs closure press				High
Proppant type—(b) factor, k vs closure press				
Well drainage radius				
Formation permeability				High
Bottom hole flowing pressure				
Pipe absolute roughness				
Proppant density				High

* some of the variables were given single point data

Then the contribution of input variance on the output was quantified, and finally variables list was done to screen out the most influencing variables/parameters. Direct benefits of data validation using uncertainty based models can be summarized as in the Table 6. Therefore, obtaining precise information on each of these parameters is very crucial for a designing a hydraulic fracturing job.

**Table 6:** Direct benefits of data validation using uncertainty based models.

	**Technical benefits**	**Cost effectiveness**
**Operating company (Client)**	i. run simple screening model in order to identify proper term of references for well stimulation (design and operation) job ii. To minimize the risk of providing inaccurate data to service companies, consultants or contractors, by identifying the critical data with the highest impact factor on the design/ operation’s outcomes	1-To save time, efforts and resources that were used to validate all data used in the treatment design by only validate the critical data. This will lead to considerable reduction in the well stimulation cost 2- To minimize the treatment designing cost by running the screening model first in order identify potential wells/formations
**Service company (implementer)**	1- To mitigate the risk in the operation by applying different scenarios in the probability based models 2- To figure out the root causes of expected and unexpected consequences during and after the application of the well stimulation treatment by applying scenarios in the probability based models	1- Reduction of the operational cost through saving time controlling the critical variables /parameters only (as per the results of the probability based simulation model) 2- Increase the success chance of the well operation by predicting the treatment outcomes for wide range of operational possibilities through the probability models
**Consultant (Designer)**	1- To give a clear decision analysis report to the client before the application of the sophisticated designing software 2- To build the treatment design on solid basis, since the design input data can be ranked and validated according to the probability based screening model. 3- To increase the design process efficiency and creditability, since no need to waste time in sensitivity analysis for uncritical parameters 4- Wide application of probability based engineering designing models will gradually allow to convert data into useful knowledge	1- To reduce the designing cost by shorten the time needed for data validation and design optimization

## 9. Conclusions

According to the results of the VBSA on the hydraulic fracturing probability-based model, it is found that the uncertainty in many variables and parameters are differently affecting the process design outcomes. In general, it is found that reservoir rock properties have insignificant effect on the fracture width compare to fracturing fluid’s effect. Moreover, the VBSA and parametric study showed that Poisson’s ratio and rock density were the major parameters that control fracture half-length, breakdown pressure and fluid’s surface injection pressure. Fracture height has been found to be predominantly controlled by formation thickness. Therefore, it is recommended to perform in-depth analysis for these factors prior conducting any designing process of hydraulic fracturing.

1. Although average fracture width that's calculated via Radial, KGD and PKN models are all show high dependency on injected fluid viscosity and injection rate, but the three models result in very different ranges of fracture average width. However, KGD model yields moderate fracture width values comparing to Radial and PKN models for the same ranges of input variables.
2. For a constant injection rate, fracture geometry is almost controlled by the injection fluid viscosity. On the other hand, it is found that uncertainty in formation rock’s properties have limited effect on the average fracture width calculation.
3. Total injected volume is found to be very sensitive to variations in fracture’s height and less sensitive to variations in leak-off coefficient and injected fluid’s viscosity. On the other hand, there is strong proportional relationship between the injected fluid’s volume with fracture’s height.
4. The propped fracture width is strong function of proppant final concentration, while variation in proppant porosity, injected fluid viscosity and formation strength are slightly affecting the calculated propped fracture width. The average fracture width is several times larger than the propped width.
5. The initial formation permeability will extremely affect the post fracturing skin factor while uncertainties in formation rock properties have minor effect on the calculated skin factor.
